# The relationship between anxiety and acute mountain sickness

**DOI:** 10.1371/journal.pone.0197147

**Published:** 2018-06-21

**Authors:** Christopher J. Boos, Malcolm Bass, John P. O’Hara, Emma Vincent, Adrian Mellor, Luke Sevier, Humayra Abdul-Razakq, Mark Cooke, Matt Barlow, David R. Woods

**Affiliations:** 1 Department of Cardiology, Poole Hospital NHS Foundation trust, Poole, United Kingdom; 2 Department of Postgraduate Medical Education, Bournemouth University, Bournemouth, United Kingdom; 3 Research Institute, for Sport, Physical Activity and Leisure, Leeds Beckett University, Leeds, United Kingdom; 4 Consultant Clinical Psychologist, Tees Esk and Wear Valleys NHS Trust, Middlesbrough, United Kingdom; 5 Defence Medical Services, Lichfield, United Kingdom; 6 James Cook University Hospital, Middlesbrough, United Kingdom; 7 Northumbria and Newcastle NHS Trusts, Wansbeck General and Royal Victoria Infirmary, Newcastle, United Kingdom; 8 University of Newcastle, Newcastle upon Tyne, United Kingdom; Texas State University San Marcos, UNITED STATES

## Abstract

**Introduction:**

Whilst the link between physical factors and risk of high altitude (HA)-related illness and acute mountain sickness (AMS) have been extensively explored, the influence of psychological factors has been less well examined. In this study we aimed to investigate the relationship between ‘anxiety and AMS risk during a progressive ascent to very HA.

**Methods:**

Eighty health adults were assessed at baseline (848m) and over 9 consecutive altitudes during a progressive trek to 5140m. HA-related symptoms (Lake Louise [LLS] and AMS-C Scores) and state anxiety (State-Trait-Anxiety-Score [STAI Y-1]) were examined at each altitude with trait anxiety (STAI Y-2) at baseline.

**Results:**

The average age was 32.1 ± 8.3 years (67.5% men). STAI Y-1 scores fell from 848m to 3619m, before increasing to above baseline scores (848m) at ≥4072m (p = 0.01). STAI Y-1 scores correlated with LLS (r = 0.31; 0.24–0.3; P<0.0001) and AMS-C Scores (r = 0.29; 0.22–0.35; P<0.0001). There was significant main effect for sex (higher STAI Y-1 scores in women) and altitude with no sex-x-altitude interaction on STAI Y-1 Scores. Independent predictors of significant state anxiety included female sex, lower age, higher heart rate and increasing LLS and AMS-C scores (p<0.0001). A total of 38/80 subjects (47.5%) developed AMS which was mild in 20 (25%) and severe in 18 (22.5%). Baseline STAI Y-2 scores were an independent predictor of future severe AMS (B = 1.13; 1.009–1.28; p = 0.04; r^2^ = 0.23) and STAI Y-1 scores at HA independently predicted AMS and its severity.

**Conclusion:**

Trait anxiety at low altitude was an independent predictor of future severe AMS development at HA. State anxiety at HA was independently associated with AMS and its severity.

## Introduction

Despite its potential fulfilment, high altitude (HA) exposure can be both physically and mentally challenging and often dangerous. Success in reaching HA in good health is known to be influenced by a number of physical factors which include the ascent mode (eg trekking/climbing vs cable car or plane) and speed, elevation gain and the ultimate altitude achieved [1,2]. There is marked inter-individual variability in the ability to cope with these challenges, such that completion rates between groups of people can vary considerably even for identical HA profiles under similar environmental conditions [1].

It is increasingly appreciated that psychological factors may be equally important to HA success, however their influence has been far less explored in the literature [3,4]. Mental wellbeing and resilience are essential for HA enjoyment and accomplishment [5]. There is some evidence to suggest a reciprocal relationship between increasing state (‘at this moment’) anxiety and HA related illnesses, whereby worsening HA related symptoms and acute mountain sickness (AMS) can lead to greater anxiety and vice versa [6,7]. There is also limited data to support a potential link between the more stable individual characteristic of trait anxiety at low altitude and the development of AMS at HA [8]. These findings are highly relevant given that anxiety affects up to 10% of young adults, who represent the greatest proportion of persons undertaking HA ventures [9].

The physical symptoms of anxiety and AMS such as headache, insomnia and fatigue frequently overlap [4,9,10]. More disabling anxiety-related symptoms such as breathlessness, hyperventilation, and increased heart rate are characteristic of significant HA exposure above 2500-3000m making distinction very challenging [1]. Differentiation of these two conditions is crucial as worsening anxiety can lead to more severe psychological problems, whereas undetected/untreated AMS can rapidly deteriorate to HA cerebral oedema, which can be life threatening [1,4,11].

Physical symptoms and somatisation at HA tend to be more severe in people with sustained background (trait) anxiety [3,10,12]. The majority of studies that have investigated the potential links between anxiety levels and AMS have tended to focus on only a few (1–2) and relatively low altitudes of <4000m [7,12–14] with limited or no adjustment for potential confounding factors such as age, gender, altitude and individual variability in the degree of hypoxia [8,15]. Studies that have examined the relationship between background trait anxiety and AMS, at terrestrial HA, have revealed contradictory findings [7,8,12–15]. They have had well recognised limitations in their methodology (post hoc diagnosis of AMS) and reporting (altitudes not stated). The published data from hypobaric and normobaric hypoxic chamber studies have been equally inconsistent [16,17].

In this study we aimed to investigate the potential links between both trait and state anxiety and AMS development (frequency and severity) over a progressive trek to very HA.

## Methods

### Design of study and participants

**Fig 1:**
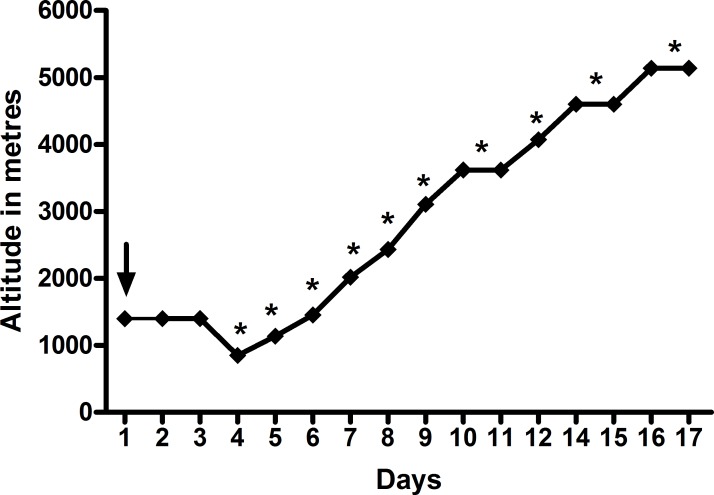
Ascent Profile and data collection time points (*). Baseline measurements (↓) were taken at 848m.

Eighty British low altitude dwelling Military servicemen undertaking a three week period of military adventure training in the Dhaulagiri region of the Himalayas were included as previously described [18]. All participants were aged >18 years and their health status required confirmation following a detailed baseline health questionnaire and a declaration of medical fitness by their General Practitioner prior to inclusion. All subjects flew to Kathmandu from the UK in consecutive groups of 6–14 staggered by two days. They spent three days of acclimatisation in Kathmandu (including arrival) before undergoing a two day staged road move to Beni (834m), where the baseline data for this study was collected. Thereafter, the participants were driven to Darbang (1030m). From there they trekked on foot over 11 days to an altitude of 5140m via a high pass at 5360m (Fig 1). Thereafter, they commenced their descent on foot to Marpha (2719m) before driving back to Kathmandu for their return to the UK. All trekking groups followed an identical ascent and exercise recovery profile with similar morning wake times. The subjects were assessed at 10 altitudes (Table 1 and Fig 1) with all assessments being performed at similar times the following morning after arrival at a new altitude. All assessments were made after the subjects had been seated for at least two minutes and prior to breakfast. The Subjects were discouraged from taking prophylactic Diamox (Acetazolamide) at the start of the trek. However, above 2500m the use of AMS related medication was permitted. All subjects underwent the same exact trek. They were all centrally catered and provided with the same meals and all treks were conducted during daylight hours.

**Table 1:** Baseline demographics.

Demographic	Overall
Number (%)	80
Men, n (%)	54 (67.5%)
Age, years (range)	32.1 ± 8.3 (19–56)
Height (cm)	173.1 ± 8.8
Weight (kg)	73.8 ± 12.2
Body mass index, kg/m2	24.4 ± 2.6
Systolic blood pressure (mmHg)	130.19 ± 16.3
Diastolic blood pressure (mmHg)	81.8 ± 15.5
Ethnicity, %	
-Caucasian	70 (87.5)
-Non Caucasian	10 (12.5%)
Smoking status (%)	

### Physiological assessments and acute mountain sickness scores

Oxygen saturations (SpO_2_) and heart rate were measured using a Nonin Onyx (Nonin Medical Inc, Plymouth, Minnesota) pulse oximeter with sampling over approximately 15 seconds. HA-related symptoms were recorded using the Lake Louis Scoring (LLS) system. This is a 5-item self -scoring questionnaire, based on the five most frequent symptoms of AMS (1 headache, 2 gastrointestinal symptoms, 3 fatigue and/or weakness, 4 dizziness/light-headedness and 5 difficulty sleeping). Each item is scored from 0 (not present) to 3 (severe). The single item scores are added up, with the total score ranging from 0–15. AMS was defined as a total LLS of ≥3 in the presence of headache and recent altitude gain. Mild AMS was defined as a LLS score of 3–5 and severe AMS a LLS ≥6, with headache and recent altitude gain for both categories [19–21]. Participants also completed a shortened 11-question version (graded from 0 [not present] to 5 [extremely severe]) of the Environmental Symptoms Questionnaire (ESQ III), known as the AMS-C score [22].

### Anxiety scores

State anxiety was quantified at all altitudes using the State-Trait-Anxiety-Inventory (STAI) [23]. This consists of 40 questions of which 20 items reflect the current anxiety state (‘at this moment’) and hence ‘State Anxiety’ (STAI Y-1) and 20 items relating to the background sustained anxiety (‘in general’) known as the Trait Anxiety (STAI Y-2). The potential answers for each of the questions are numbered 1–4 on a Likert Scale 1 ‘almost never’, 2 ‘sometimes’, 3 ‘often’ and 4 ‘almost always’. The total score ranges from 20–80 for each of the STAI Y-1 and Y-2 questionnaires respectively, with higher scores indicating greater anxiety. A cut point of >39–40 has been suggested to detect clinically significant state anxiety in younger adults [24,25]. STAI Y-1 scores were assessed on all subjects and STAI Y-2 scores on 50 subjects at 848m.

### Ethics

All subjects were provided with a written participant information sheet >48 hours prior to inclusion into the study. All participation was voluntary and all subjects underwent detailed written informed consent before inclusion. The study was approved by the Ministry of Defence Research and Medical Ethics Committee (MODREC) and was conducted according to the standards of the Declaration of Helsinki.

### Statistical analysis

Data were analysed using GraphPad InStat version 3.05 and SPSS^®^ statistics version 22 with all graphical figures presented using GraphPad Prism version 4.00 for Windows (GraphPad Software, San Diego, CA, USA). Sample size calculations were performed using a proprietary determined sample- size calculator (GraphPad StatMate version 2.00 for Windows). Data inspection and the Kolmogorov-Smirnov test was undertaken to assess normality of all continuous data, which were presented as mean ± standard deviations. Categorical variables were compared using Chi-Squared tests. Comparison of unpaired data was performed using an unpaired T test and a Mann-Whitney Test for parametric and non-parametric data respectively. Correlations were performed using Pearson and Spearman rank correlation (±95% confidence interval, CI) for parametric and non-parametric data respectively. A Factorial two-way repeated measures ANOVA was undertaken to assess the effects of sex and altitude and their potential interaction on potential changes in STAI Scores at HA.

The independent predictors of increasing STAI Scores were investigated using multiple linear regression analysis. Conditional stepwise binary logistic regression analysis was performed to investigate the independent significant anxiety (STAI Y-1>39) and AMS. Only variables with p-values of <0.25 on univariate correlation, t-test and Chi-square tests were entered into the regression models. Results were reported as the odds ratio (*B*) and 95% confidence interval as well as the overall model fit (r^2^). Collinearity (Pearson's correlation coefficient >0.7) between variables was tested before modeling, and if present, only 1 variable was entered into the binary logistic regression test. Statistical significance was defined as a p-value of <0.05.

### Sample size and power calculation

In a previous study of 44 subjects, Oliver et al observed a significant increase in anxiety defined using a shorted version of the STAI scale with an AMS affecting 43% of subjects at >2476m [3]. Based on published report STAI Scores in healthy young adults we estimated that a sample size of 50 subjects would provide ≥80% power to detect a paired difference between mean STAI Scores of ≥1.50 with a significance level (alpha) of 0.05 (two-tailed) [26].

## Results

### Subject demographics

The average age of the 80 participants was 32.1 ± 8.3 (range 19–56) (Table 1). We included 54 men (67.5%) and 26 (32.5%) women who were of similar age (32.3±8.4 vs 31.6±8.4; p = 0.71) (Table 1). The number of subjects in the consecutive trekking groups was 14 (team 1), 10 (team 2), 6 (team 3), 9 (team 4), 11 (team 5), 12 (team 6), 12 (team 7) and 6 (team 8) respectively. No subjects took Diamox (Acetazolamide) below 2430m.

### Effect of HA on physiological variables and STAI/AMS scores

**Fig 2:**
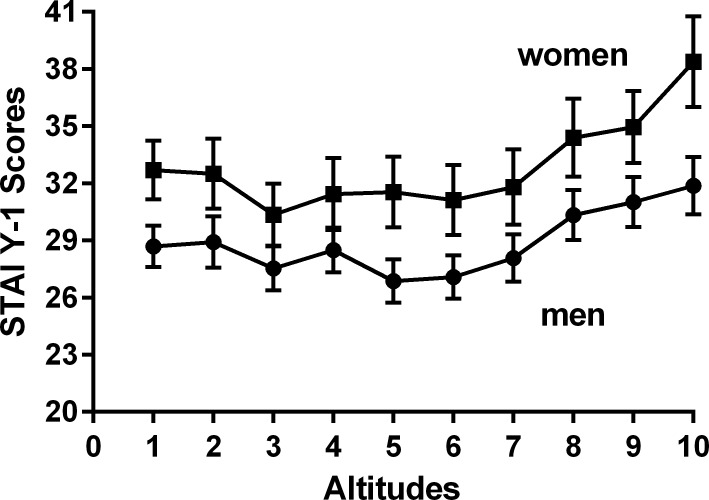
Comparative changes in STAI Y-1 Scores at each altitude in men versus women.

Increasing HA led to an increase in LLS, AMS-C scores, heart rate and a reduction in SpO_2_ (Table 2). STAI Y-1 Scores initially fell from 848m-3619m, before significantly increasing at ≥4072m (F = 3.1; df 9–558: p = 0.01) (Table 2). At the baseline study altitude of 848m, STAI Y-1 Scores were significantly higher in women than men (32.7±7.9 vs 27.5±8.1; p = 0.001) (Fig 2). Overall (10 altitudes) STAI Y-1 Scores remained higher in women versus men (32.9 ± 9.5 vs 27.1; p<0.0001) and significantly differed among the eight trekking groups and ranged from 24.6±6.9 (team 5) to 41.2±8.5 (team 8) (p <0.0001), but were not influenced by the size of the trekking group. On factorial two-way Repeated Measures ANOVA there was a significant effect for female sex (F = 3.97; p = 0.002), and altitude (F = 7.95; p = 0.006) with no interaction between sex x altitude (F = 1.6; p = 0.16) on STAI Y-1 scores.

**Table 2:** Changes in Physiological parameters, Acute Mountain sickness and anxiety scores.

Altitudes	848m	1179m	1456m	2017m	2430m	3107m	3619m	4072m	4600m	5140m	P value
	(1)	(2)	(3)	(4)	(5)	(6)	(7)	(8)	(9)	(10)	
SpO2,%	97.0±1.6	96.7±2.3	96.6±1.8	96.0±2.4*	94.8±3.9*	93.0±4.3*	91.9±3.8*	86.5±5.6*	82.8±5.8*	80.7±5.4*	<0.0001
Heart rate/minute	71.5±11.6	72.1±13.2	71.6±12.5	68.3±12.0*	68.6±12.5*	69.1±12.5*	70.4±11.5	77.5±11.9*†	79.6±14.6*†	81.2±15.4*†	<0.0001
Lake Louise Score	0.80±1.9	0.78±1.7	0.53±1.1	0.68±1.1	0.58±1.4	0.73±1.3	1.30±1.9*	1.50±2.0*	1.7±1.9*	1.8±2.2*	<0.0001
AMSC Score	1.6±5.3	1.2±4.7	1.1±4.4	0.60±1.6	1.0±5.2	1.0±1.8	2.0±4.43ǂ	3.2±5.7*ǂ	2.8±4.3*ǂ	4.1±7.3*ǂ	0.001
AMS, n (%)	0/80	0/80	0/80	1/80	1/80	3/79	9/79	11/78	18/77	19/70	<0.0001
	0%	5.0%	0.0%	1.3%	1.3%	3.8%	11.4%	14.1%	23.4%	27.5%	
Severe AMS	0/80	1/80	0/80	0/80	1/80	1/79	5/79	3/78	8/77	6/70	<0.0001

Significant Post-test pair wise comparisons

*versus baseline

† versus altitudes 3, 4, 5 and 6

ǂ vs 4.

### Relationship between STAI scores to LLS/AMS-C scores and physiological variables

STAI Y-1 and STAIY-2 Scores at 848m were strongly correlated (n = 50 pairs; r = 0.82; 0.70–0.90: p<0.0001). STAI Y-1, but not STAI Y-2 scores at 848m correlated with LLS (r = 0.33; 0.11–0.51; p = 0.003) and AMS-C Scores (r = 0.36; 0.14–0.54; p = 0.001). Over the 10 altitudes examined STAI Y-1 scores positively correlated with LLS (r = 0.31; 0.24–0.3; P<0.0001), AMS-C Scores (r = 0.29; 0.22–0.35; P<0.0001) and inversely correlated with age (r = -0.18; p<0.0001) and SpO_2_ (r = -0.08; p = 0.03). The other significant univariate predictors of an increase in STAI Y-1 scores were trekking group and female sex.).

On Multiple linear regression analysis, entering these prediction variables along with altitude (p = 0.10 on univariate analysis), showed that the independent predictors of increasing STAI Y-1 scores (dependent variable) were trekking team, female sex, younger age, increasing heart rate and both LLS and AMS-C Scores (overall r^2^ = 0.26; p<0.0001). In Binary regression analysis the independent predictors of significant state anxiety (STAI Y-1 >39) were female sex, trekking team (earlier groups were a stronger predictor), lower age, higher heart rate, and increased LLS or AMS-C Scores (r^2^ = 0.29; p<0.0001).

### Prevalence of AMS

A total of 38/80 subjects (47.5%) developed AMS, which was mild in 20/80 (25%) subjects and severe in 18/80 (22.5%). The prevalence of any AMS varied by trekking group affecting 6/14 (42.9%), 8/10 (80%), 5/6 (83.3%), 4/9 (44%), 5/11 (45.55%), 4/12 (33%), 4/12 (33%) and 2/6 (33%) of the 8 consecutive trekking groups (Chi Squared test for trend p = 0.07). The future risk of developing both AMS (23/38, 60.5% vs 15/42 = 35.7%; p = 0.04) and severe AMS (13/38, 34.2% vs 5/42, 11.9%; p = 0.03) at HA was higher among individuals who were in the first four groups versus the second respectively. Over the entire 10 altitude study time points (ie total occasions), AMS prevalence significantly varied by trekking group occurring in 11/135, 19/98, 8/58, 11/88, 9/109, 5/109, 4/119 and 3/55 groups respectively (p = 0.001). Furthermore, AMS (49/379 vs 21/402; OR 2.7; 1.58–4.59: p = 0.0002) and severe AMS (20/369 vs 8/412; Odds ratio 2.90: p = 0.01) was more common among the first four versus the second four trekking groups.

### Relationship between STAI scores and AMS

There were no significant differences in STAI Y-1 (27.5±7.3 vs 30.8±9.3; p = 0.10) or STAI Y-2 (31.2 ± 6.8 vs 33.5 ± 8.1; p = 0.28) scores at 848m (baseline) between participants who went on to developed any AMS versus those who did not respectively. However, trait (STAI Y-2) anxiety (31.2±6.6 vs 37.5 ±9.7; p = 0.03) but not state (STAI-Y-1) anxiety (28.8± 8.1 vs 30.1±9.5; p = 0.63) scores, at 848m, were significantly higher in those who subsequently developed severe AMS at HA. Across the 10 altitudes STAI Y-1 scores were on average higher in those with both AMS (37.8±9.2 vs 28.1±8.7; P<0.0001) and severe AMS (40.3±10.5 vs 28.6±8.9; P<0.0001) versus those without respectively.

### Independent predictors of AMS

The univariate predictors of AMS were female sex, SpO_2_, STAI Y-1 Scores, altitude and heart rate (Table 3). These variables along with height and weight (p<0.25 on univariate analysis) were entered into the binary logistic regression with AMS as the dependent variable. Increasing STAI Y-1 scores, altitude, heart rate and reducing SpO_2_ were independent predictors of AMS (overall r^2^ = 0.41; p<0.0001) (Table 4). The univariate predictors of severe AMS were STAI Y-1 Scores, altitude, heart rate and SpO_2._ Entry of these variables along with height and weight (p<0.25 on univariate analysis) revealed that STAI Y-1 scores were the only independent predictor of AMS (overall r^2^ = 0.32; p<0.0001) (Table 4). STAI scores remained as an independent predictor of both AMS and separately severe AMS on sensitivity analyses, using differing entry variable p values (p<0.05–0.25). This significant prediction remained even if trekking group, height and weight or altitude were removed from the analysis. A STAI Y-1 >39 was an even stronger independent predictor of AMS (*B* = 7.9; 3.9–16.1; P<0.0001) and severe AMS (*B* = 6.8; 2.70–16.9; p<0.0001).

**Table 3:** Univariate predictors of acute mountain sickness at high altitude.

	No AMS	AMS	P value
Age, years	32.1 ± 8.4	31.3 ± 6.7	0.81
Sex (%)			
-Men	68.9%	51.6%	
-Women	31.1%	48.4%	0.007
Height, cm	173.2 ± 8.9	171.2 ± 7.8	0.09
Weight, kg	73.7 ± 12.2	71.7 ± 10.9	0.14
Body mass index, kg/m^2^	24.4±2.6	24.4±2.3	0.76
Caucasian (%)	89.8%	90.5%	0.98
Current smoker (%)	7.3%	11.1%	0.47
Altitude, m	2760±1793	4330±827	<0.0001
Heart rate/minute	72.1±13.0	80.8±15.6	<0.0001
SpO_2_, %	92.6±6.2	83.1±8.3	<0.0001

**Table 4:** Independent predictors of acute mountain sickness at high altitude.

	Odds ratio (B)	95% CI for Odds ratio (B)		P value
		Lower	Upper	
**AMS**				
Altitude	1.38	1.1200	1.69	0.002
Trekking group	0.78	0.68	0.90	<0.0001
SpO_2_	0.92	0.87	0.97	<0.001
STAI Y-1 Score	1.11	1.07	1.15	<0.0001
**Severe AMS**				
Altitude	1.31	0.97	1.76	0.04
Trekking group	0.83	0.68	1.00	0.06
SpO_2_	0.93	0.86	1.00	0.07
STAI Y-1	1.13	1.07	1.18	<0.0001

STAI Y-1 scores were also a univariate and independent predictor of AMS at the next higher altitude (STAI Y-1 *B* = 1.04; 1.0–1.07; p = 0.003; overall r^2^ = 0.24). STAI Y-2 trait anxiety scores (*B* = 1.14; 1.006–1.28; p = 0.04), measured at 848m, were an independent predictor of future severe AMS, after adjusting for age and sex and trekking group (overall; r^2^ = 0.23) and remained consistent even after removing trekking group from the analysis.

## Discussion

In this study trait (STAI Y-2), but not state (STAI Y-1) anxiety, measured at 848m was an independent predictor of future severe AMS at higher altitudes. Overall, across the 10 study altitudes increasing STAI Y-1 scores were independently linked to AMS and its severity. Independent predictors of significant state anxiety (STAI Y-1 >39), were female sex, trekking group, younger age, higher heart rate, and increasing AMS Scores.

Increasing HA exposure triggers a number of recognised physiological responses which have the potential to affect mood and increase anxiety. There is widespread sympathetic activation which leads to an increase in resting heart rate and greater predisposition to symptomatic palpitations [2,6,21]. Ambient hypobaric hypoxia at HA leads to compensatory hyperventilation to maintain adequate tissue oxygenation [1]. These normal physiological responses can be misinterpreted as a sign of pathology in some individuals and the potential to trigger anxiety and promote its escalation [4]. With increasing altitude, HA related symptoms such as fatigue, breathless and insomnia are more common and are potential triggers to increasing stress and potential anxiety [1,27]. Additional situational and environmental factors such as social isolation, lack of familiarity and the often intense cold can be further pro-anxiety triggers, particularly in those with greater vulnerability [4]. Conversely, there is evidence that worsening anxiety in itself can lead to greater somatisation and physical symptoms at HA an increased susceptibility to AMS [6]. Our data supports this. We found that increasing STAI Y-1 symptoms were linked to greater risk of AMS and its more severe forms. It is interesting that trait STAI Y-2 scores and not state STAI Y-1 scores, taken at the study baseline altitude of 848m, were predictive of future risk of severe AMS. This is perhaps not surprising, given that susceptibility to AMS appears to be a relatively stable individual trait with increasing evidence for a genetic predisposition that can be influenced by additional factors such as ascent profile [28–30]. We found that trait anxiety and STAI Y-2 questions, as opposed to STAI Y-1 was a predictor of future AMS given its representation of more stable individual trait anxiety levels [23].

A number of previous studies have investigated the potential links between trait anxiety and risk of AMS and HA-related headache. In one of the prospective studies to report this potential link, Missoun et al (1992) studied 100 subjects (80 men and 20 women) travelling to above 3500m in the Himalayas [8]. They found that those who were more susceptible to AMS had higher pre-expedition trait anxiety (STAI Y-2) scores, and higher anxiety, when asked to imagine starting out on a high altitude ‘summit day’. There was no difference between the AMS susceptible and non AMS susceptible groups, on a pre expedition measure of behavioural adaption to stress (Bortner scale), nor on a pre-expedition measurement of state anxiety (STAI Y-1) [31]. In another study, Bian et al studied 285 Chinese men at 400m and within 24 hours of ascent to 3450m over a four day trek [13]. They found that persons who developed AMS (LLS ≥3 with headache) had higher scores for baseline somatization, obsession–compulsion, depression, anxiety and hostility on the nine factor Symptoms Checklist-90 (SCL-90). However, on multivariate analysis only somatization remained the only independent baseline predictor of AMS [13]. In a follow up study by the same author, using a similar ascent protocol, Bian et al (n = 163) observed that subjects with AMS exhibited greater anxiety using the Self-Rating Anxiety Scale (SAS). However, SAS scores were not found to be an independent predictor of AMS [14]. In a recent, albeit smaller study (n = 44) over a near identical trek to ours, in the Dhaulagiri region of Nepal, Oliver et al noted that total AMS Scores were linked to increased levels of anxiety, quantified using the Short Form (six items, 0–3 Likert scale) STAI score. This score, however, failed to predict future AMS on following day. Conversely, we found that STAI Y-1 scores were predictive of AMS at the next altitude supporting the association with AMS [3]. Dong et al studied 426 men who ascended from near sea level to 3600m [12]. They found that the SAS scores was marginally, yet significantly higher in the 149 individuals (35%) who developed AMS versus those without (48.0 vs 45.4; p<0.001). However, they did not assess the independent predictors of AMS separately.

One of the strengths of our study was its relatively large sample size for an extreme HA study and that we studied the participants over 10 consecutive altitudes during a recognised trek. The majority of previous studies tended to be smaller and assessed only 1–2 altitudes. Furthermore, the subjects in our study all underwent an identical ascent protocol, over 8 trekking teams, staggered by two day intervals. We included much higher altitudes than most of the studies cited above, which could have acted to strengthen the relationship between anxiety and AMS given that LLS, AMS and STAI scores were far greater at the higher altitudes. We observed a significant reduction in STAI Y-1 scores from 848 to 3107m followed by a significant rise to above baseline (840m) values from ≥3619m. LLS and AMS-C scores and the AMS burden similarly increased from 3619m too. The highest average STAI scores were at 5140m.

The independent predictors of significant anxiety in this study, defined as a STAI Y-1 >39 included younger adults, female sex, higher heart rate and a greater burden of AMS related symptoms. This suggests that increasing STAI scores were influenced by HA-related factors (increasing heart rate). It is well reported that anxiety levels tend to be higher in younger adults and are generally greater in women [32,33]. This is, to the author’s knowledge, the first study to confirm this previous observation at HA.

Whilst the LLS and AMS-C refer largely to physical symptoms (eg headache and gastrointestinal upset) and the STAI Y-1 questionnaires to ‘mental symptoms’ the clinical features of anxiety and AMS overlap. Hyperventilation and increased heart rate, fatigue, muscle ache and insomnia, gastrointestinal upset and headaches which are well recognised features of generalised anxiety are well reported effects of HA exposure alone. LLS, AMS-C and heart rate all represent transient and ‘state’ measurements and hence are more likely to relate to ‘state’ rather than trait anxiety and this is supported by our data [19–21]. Our study is strengthened by the fact that we examined not only all AMS but also its more severe forms and both state and trait anxiety. The fact that we were able to observe a significant link between anxiety scores on multivariate analysis and following sensitivity testing strengthens our findings. Unfortunately, the nature of this relationship remains elusive. Whilst we have not been able to determine a causative link between anxiety and AMS, our observation that rising anxiety precedes worsening AMS-related symptoms tend to support this. Conversely, the relationship may be more reciprocal with rising AMS symptomology and anxiety accelerating one another.

We found that both STAI Y-1 Scores and AMS burden appeared to be independently influenced by the trekking group order rather than the size of the trekking group ‘per se’. The higher burden of AMS in the earlier first four versus the second four groups is an interesting observation. Baseline allocation to the first four trekking groups was linked to an increased risk of future AMS and severe AMS strengthening this finding, and making a chance effect less likely. The reasons for these differences in STAI Y-1 scores and AMS burden may relate to differences in group dynamics and behaviour, coupled with the fact that the trekking success of the earlier groups would have filtered back to the later trekking groups to lessen their concerns.

This study has a number of limitations that should be acknowledged. HA-related symptom scores, STAI questionnaires and physiological measurement (eg SpO_2_ and heart rate) were all undertaken at a similar time together which was in the early morning, pre breakfast and >12 hours after arrival at each altitude, the afternoon before. Hence, we cannot exclude the fact that participants LLS/AMS-C and STAI Y-1 Scores could have been significantly different the evening before and within hours of arrival at a respective altitude, compared with the measurements obtained during the morning assessments. Baseline STAI Y-2 and HA scores were measured at 848m in Nepal and not at sea level. This was the lowest feasible altitude to obtain these measurements, given the geography in Nepal and our desire to undertake all key assessments within the main expedition timeframe to allow meaningful comparisons between scores. We did not explore the effects of STAI Scores on measures of cognition, self-motivation and depression which could have been affected by the levels of anxiety.

## Conclusions

Trait anxiety at low altitude was an independent predictor of future severe AMS development at HA. State anxiety, was independently associated with AMS and its severity. A future prospective study investigating the effects of anxiety reduction interventions on AMS development would be helpful, as would further work on the factors jointly and separately contributing to state anxiety and AMS.

## Supporting information

S1 File Anonymised data file for the study. (CSV)
